# Predictive criteria for the development of intra-abdominal hypertension and abdominal compartment syndrome

**DOI:** 10.1186/cc13359

**Published:** 2014-03-17

**Authors:** D Lyer, P Rastogi, A Aneman, S D'Amours

**Affiliations:** 1 Liverpool Hospital, Sydney, Australia

## Introduction

This study aims to develop predictive criteria to identify which patients are at risk of developing intra-abdominal hypertension (IAH) and abdominal compartment syndrome (ACS) upon admission to the ICU.

## Methods

This is a prospective, observational study of403 consecutively admitted ICU patients in a 3-month period, requiring the insertion of an indwelling urinary catheter. Intra-abdominal pressure was measured at least twice daily in all patients.

## Results

Thirty-nine and 2% of patients developed IAH and ACS as per consensus definitions. Upon ICU admission, patients that would go on to develop IAH had a significantly higher APACHE III score (62 (44 to 81) vs. 50 (37 to 68); *P *< 0.001), abbreviated SOFA score (6 (4 to 8) vs. 4 (2 to 6); *P *< 0.001), CVP (17 (13 to 20) vs. 15 (12 to 17) mmHg; *P *< 0.001), lactate (2.2 (1.4 to 3.8) vs. 1.5 (1.1 to 2.4) mmol/l; *P *< 0.001), INR (1.3 (1.1 to 1.5) vs. 1.2 (1.1 to 1.3); *P *< 0.001), bilirubin (11 (7 to 19) vs. 10 (6 to 15) μmol/l; *P *= 0.027), creatinine (108 (76.8 to 175) vs. 84.0 (66.0 to 118) μmol/l; *P *< 0.001), 24-hour fluid balance (2.47 (0.95 to 4.05) vs. 1.23 (0.29 to 2.27) l; *P *< 0.001), vasopressor requirement (60 vs. 39%; *P *< 0.001), mechanical ventilation requirement (71 vs. 58%; *P *= 0.011), PEEP (8.00 (5.00 to 10.00) vs. 5.50 (5.00 to 8.00) cmH_2_O; *P *< 0.001), and peak airway pressure (24 (21 to 30) vs. 22 (19 to 24) cmH_2_O; *P *< 0.001). These patients also had a significantly lower abdominal perfusion pressure (61 (55 to 71) vs. 69 (60 to 77) mmHg; *P *< 0.001), pH (7.29 (7.22 to 7.35) vs. 7.33 (7.28 to 7.37); *P *< 0.001) and PaO_2_:FiO_2 _ratio (182 (100 to 263) vs. (264 (168 to 371); *P *< 0.001) upon ICU admission. Abdominal distension (odds ratio, 4.95; 95% CI, 1.19 to 7.24; *P *< 0.001), hemoperitoneum/ pneumoperitoneum/intraperitoneal fluid collection (odds ratio, 3.61; 95% CI, 1.29 to 10.12; *P *= 0.014), obesity (odds ratio, 3.41; 95% CI 1.97 to 5.90; *P *< 0.001), fluid received >2.3 l (odds ratio, 2.68; 95% CI, 1.48 to 4.84; *P *= 0.001), abbreviated SOFA score >4 points (odds ratio, 2.49; 95% CI, 1.49 to 4.15; *P *< 0.001) and lactate >1.4 mmol/l (odds ratio, 2.28; 95% CI, 1.33 to 3.91; *P *< 0.001) were identified as independent predictors of IAH upon admission to ICU. The presence of three or more of these risk factors at admission predicted the onset of IAH with sensitivity of 75% and a specificity of 76%, and the onset of grade II, III and IV IAH with a sensitivity of 91% and a specificity of 62%.

## Conclusion

IAH is a common clinical entity in the intensive care setting. Predictive criteria, based on data readily available upon a patient's admission to ICU, were developed and effectively predicted the risk of developing IAH.

